# Mechanical and Gravimetric Response of Surgical Suture Biomaterials to Commercial Mouthrinses During Static In Vitro Exposure

**DOI:** 10.3390/bioengineering13101178

**Published:** 2026-10-09

**Authors:** Alicja Choszczyk, Anna Ciołko, Wiktoria Nowak, Leszek Szalewski, Ewelina Kosicka, Żaneta Anna Mierzejewska

**Affiliations:** 1 Institute of Biomedical Engineering, Faculty of Mechanical Engineering, Bialystok University of Technology, Wiejska 45C, 15-351 Bialystok, Poland; 86275@student.pb.edu.pl (A.C.); 86971@student.pb.edu.pl (A.C.); 86288@student.pb.edu.pl (W.N.); 2 Digital Dentistry Lab, Department of Dental and Maxillofacial Radiodiagnostics, Faculty of Medical Dentistry, Medical University of Lublin, 20-059 Lublin, Poland; leszek.szalewski@umlub.edu.pl; 3 Department of Computerization and Robotics of Production, Faculty of Mechanical Engineering, Lublin University of Technology, Nadbystrzycka 36, 20-618 Lublin, Poland; e.kosicka@pollub.pl

**Keywords:** surgical suture biomaterials, oral biomaterials, mouthrinses, material–environment interactions, maximum breaking force, aqueous aging, wet-state mass change, polymeric biomaterials

## Abstract

Surgical sutures are temporary load-bearing biomaterials whose performance in the oral cavity may be altered by postoperative mouthrinses. Seven USP 4-0 suture materials were immersed in four commercial mouthrinses for 3, 7, 10, and 14 days in a balanced factorial design (*n* = 3 per condition), with artificial saliva as a time-matched reference. Maximum breaking force (Fmax), apparent wet-state mass change, and solution pH were evaluated, and Day 14 re-dried mass was determined in an additional cohort (*n* = 5). Fmax was significantly affected by material, mouthrinse, time, and all interaction terms (*p* < 0.001), with material showing the largest main effect (partial η^2^ = 0.957). Mean Fmax decreased by 57.1% for PGA and 53.3% for PGLA between Days 3 and 14 when averaged across mouthrinses. PGLA also declined in artificial saliva (−49.1%), showing that temporal loss was not exclusive to mouthrinse exposure. Mean apparent wet-state mass change ranged from 85.1% for silk to 8.5% for nylon 66 and showed a weak pooled association with Fmax (r = −0.163, *p* = 0.086); suture-related pH shifts were small. Under static immersion, response was specific to the material–formulation pair and evolved with time. Continuous immersion without medium renewal or prior salivary conditioning does not reproduce intermittent clinical mouthrinse use.

## 1. Introduction

Surgical sutures are temporary load-bearing biomaterials that must maintain wound approximation while the surrounding tissue regains mechanical integrity. In the oral cavity, they function at a continuously hydrated biomaterial–fluid interface exposed to saliva, ions, temperature variations, microbial products, tissue movement, and functional loading. Clinically relevant suture performance therefore depends not only on initial breaking force but also on the stability of the material–environment system during early healing [1,2,3,4,5,6].

Suture behavior emerges from coupled material and structural variables. Polymer chemistry governs hydration and hydrolytic susceptibility, whereas filament architecture, surface characteristics, coating, capillarity, and knot geometry influence load transfer and local stress concentration [1,2,5,7,8,9,10]. Synthetic absorbable sutures based on hydrolytically degradable polymers, such as polyglactin, polyglycolic acid, and polydioxanone, may undergo water penetration, hydrolytic chain scission, molecular-weight reduction, and progressive loss of load-bearing capacity, whereas non-absorbable materials can still exhibit hydration-related changes in deformation, filament interaction, and knot mechanics [11,12,13,14,15,16]. Conventional categories such as absorbable/non-absorbable or mono-/multifilament are therefore clinically useful but do not fully describe functional stability under aqueous exposure.

Postoperative mouthrinses introduce an additional environmental variable. Commercial formulations differ in pH and may contain fluoride species, essential oils, antiseptics, surfactants, solvents, and preservatives capable of interacting with polymeric biomaterials. Chlorhexidine- and essential-oil-containing mouthrinses have been shown to alter suture mechanics, but the magnitude and direction of the response depend on material type, gauge, and exposure conditions [17,18,19,20]. Studies of beverages and toothpastes likewise show that environmental effects cannot be generalized across exposure media [21,22,23,24]. The relevant engineering question is therefore not whether a mouthrinse is intrinsically “aggressive”, but how a specific material responds to a specific chemical environment over time. Existing laboratory evidence supports investigating mechanical compatibility but does not show that changes observed under prolonged immersion cause clinical wound failure.

Most mouthrinse–suture studies have focused on tensile or breaking-force outcomes in relatively limited material sets [17,18,19,20]. Mechanical testing alone does not reveal whether changes are accompanied by hydration, liquid retention, swelling, adsorption, deposition, or shifts in the surrounding chemical environment. Parallel mechanical, gravimetric, and bulk-pH measurements describe complementary aspects of the exposure response, although none directly identifies surface reactions. A factorial design that varies material, medium, and time simultaneously allows main effects to be separated from material-specific interactions.

The present study used a balanced 7 × 4 × 4 design to compare seven USP 4-0 surgical suture biomaterials exposed to four commercial mouthrinses over 14 days. Maximum breaking force (Fmax), apparent wet-state mass change, and pH were evaluated in parallel, with specimen-free blank controls used to separate material-related pH changes from spontaneous solution drift. Re-dried mass was additionally determined at Day 14 to separate net nonvolatile mass change from mass removed by rinsing and drying, and artificial saliva served as a time-matched reference medium. The primary hypothesis was that Fmax would be governed by interacting effects of material, mouthrinse, and exposure time rather than by any single factor. Secondary, exploratory objectives were to examine the association between apparent wet-state mass change and Fmax and to determine whether the sutures altered the bulk pH of the media.

## 2. Materials and Methods

### 2.1. Suture Biomaterials and Factorial Study Design

Seven commercially available surgical sutures with different polymer compositions and filament architectures were included (Table 1): nylon 66 (NL), polypropylene (PP), polyester (PB), polyglactin (PGLA), polyglycolic acid (PGA), silk (SI), and polyamide (SB). All had a nominal USP size of 4-0 and a nominal length of 75 cm. NL, PP, PB, PGLA, and PGA were manufactured by Shandong Haidike Medical Products Co., Ltd. (Heze, China) and supplied under the RT MED brand; SI and SB (marketed as Supramid) were manufactured by LUX Sutures (Weiswampach, Luxembourg). The set was selected to span distinct polymer chemistries, absorbability classes, and filament constructions relevant to oral wound closure. Before immersion, sutures were removed from their sterile packaging, separated from the needles, and cut into 15 cm specimens. Multiple boxes of each suture type were purchased to provide sufficient individually packaged sutures for specimen preparation. The specimens came from the same manufacturing batch of the corresponding manufacturer. Package identity was not included as an analytical factor, and between-batch variability was not assessed.

**Figure 1:**
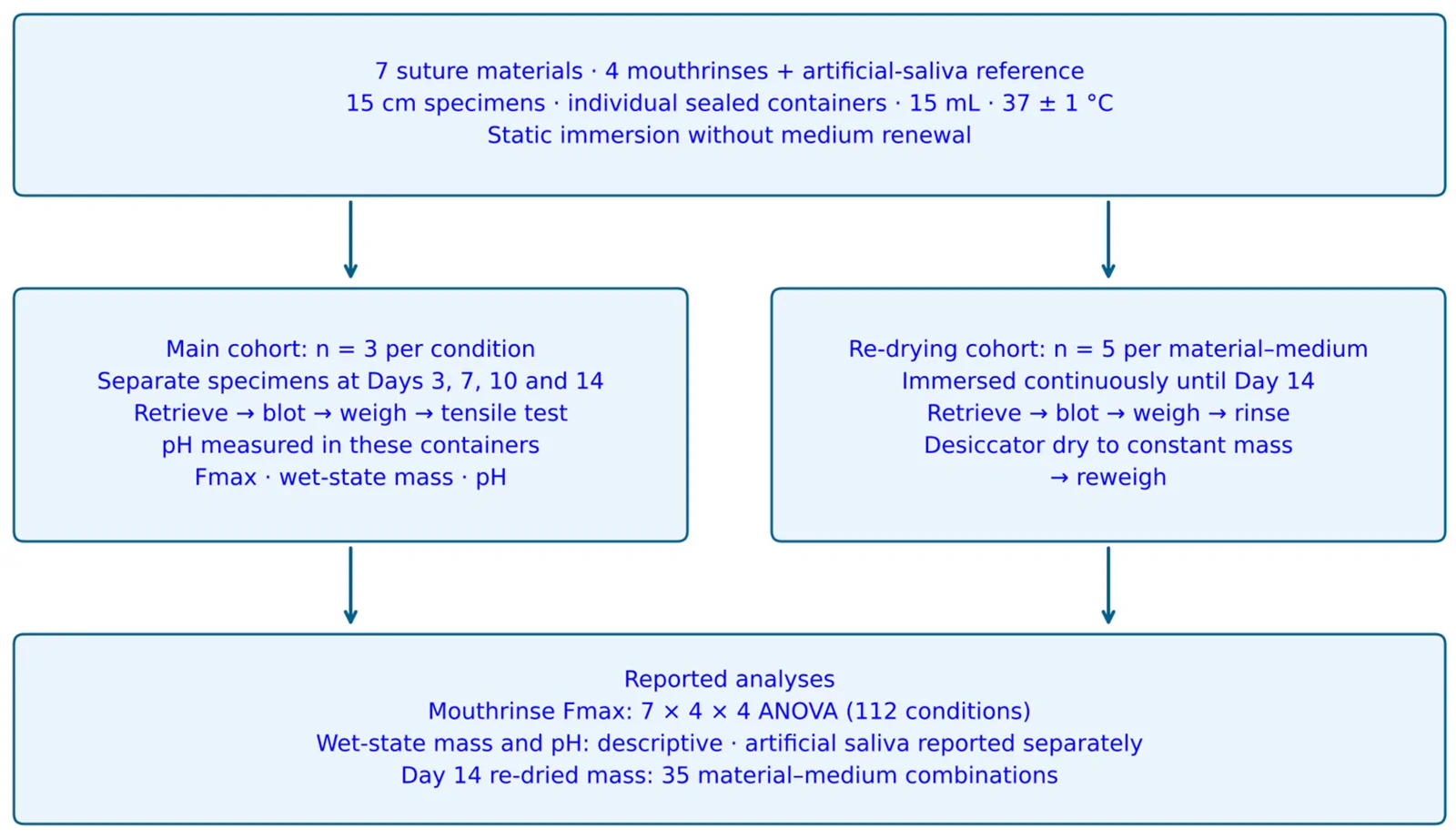
Specimen allocation and measurement workflows. At each time point, three specimens per condition were weighed, tensile-tested, and used for pH measurement; a separate re-drying cohort (*n* = 5 per material–medium combination) was immersed until Day 14 and then rinsed and dried. The commercial-mouthrinse factorial ANOVA comprised 112 conditions; artificial saliva was analyzed as a separate reference.

The main factorial design comprised seven materials, four mouthrinses, and four exposure times (Days 3, 7, 10, and 14), giving 112 conditions. For each material–medium–time condition, three separate specimens were retrieved at the assigned time, blotted, weighed, and tensile-tested, and the pH of their containers was measured; these specimens therefore provided the wet-state mass, pH, and Fmax data. Specimens allocated to different time points were separate, and no specimen was loaded twice. In addition, a re-drying cohort of five specimens per material–medium combination was placed in the incubator at the start of the experiment under the same conditions and retrieved at Day 14 for wet weighing, rinsing, and desiccator drying (Figure 1). Artificial saliva was tested as an additional reference medium for the same seven materials and time points; its results are reported separately from the 7 × 4 × 4 mouthrinse ANOVA. Re-dried mass was therefore determined at Day 14 only (35 material–medium combinations, including artificial saliva).

### 2.2. Mouthrinses

**Figure 2:**
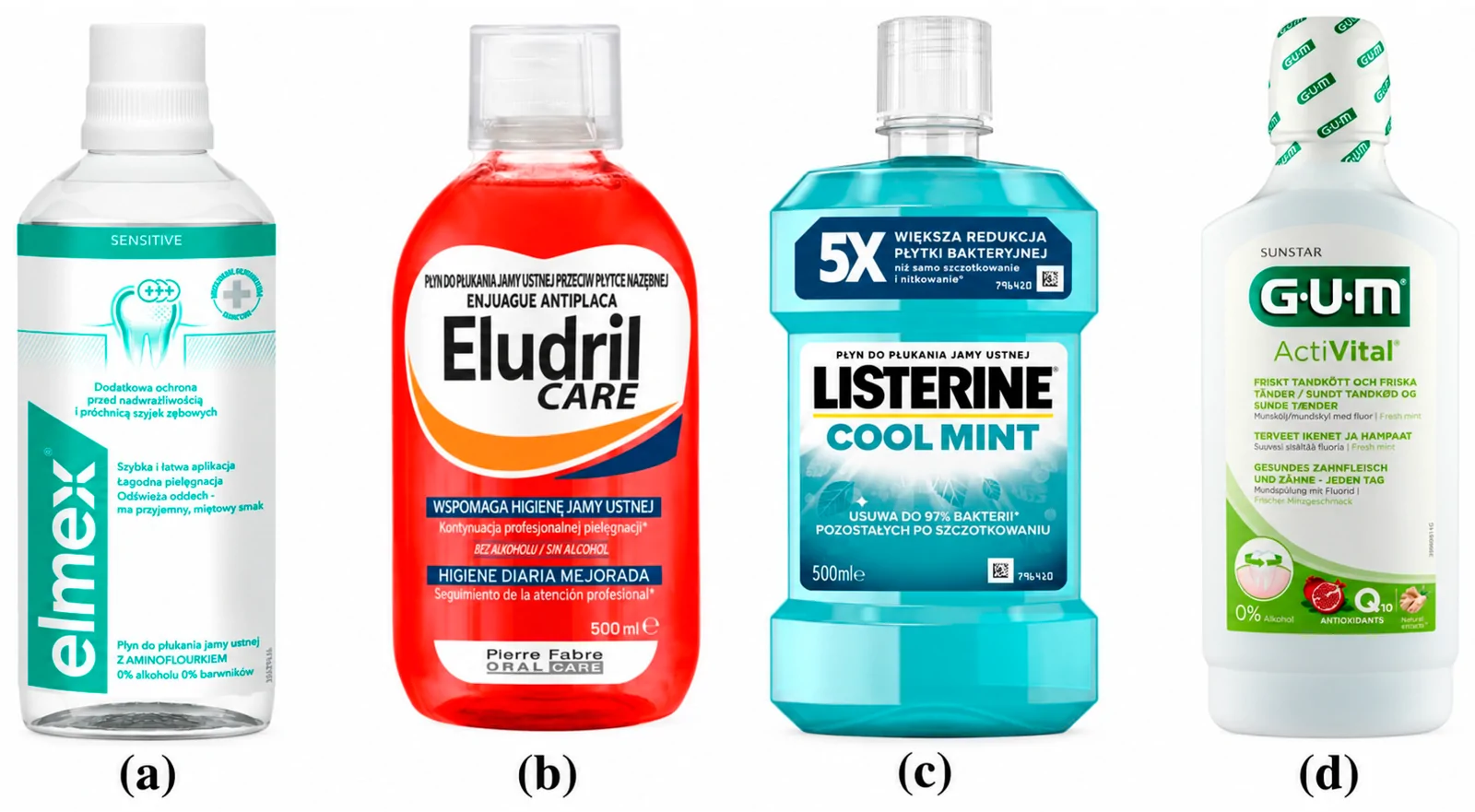
Commercial mouthrinses used as exposure media: (**a**) Elmex Sensitive, (**b**) Eludril Care, (**c**) Listerine Cool Mint, and (**d**) GUM ActiVital Fresh Mint.

Four commercial mouthrinses were tested (Table 2, Figure 2): Elmex Sensitive (olaflur and sodium fluoride, 250 ppm total F; Colgate-Palmolive, Warsaw, Poland), Eludril Care (0.05% chlorhexidine digluconate and 0.05% cetylpyridinium chloride; Pierre Fabre Médicament, Lavaur, France), Listerine Cool Mint (essential oils and 21.6% ethanol; JNTL Consumer Health, Warsaw, Poland), and GUM ActiVital Fresh Mint (sodium fluoride, 248 ppm F, and ethyl lauroyl arginate, LAE; Sunstar Europe S.A., Etoy, Switzerland). Elmex, Eludril, and GUM were ethanol-free. Measured initial pH values were 4.52, 5.43, 4.11, and 5.53, respectively. Manufacturer information supports the general formulation characteristics [25,26,27,28] but does not establish the INCI composition of a specific batch; batch labels remain the authoritative source, as marketed formulations may vary by region and revision.

Ingredient overlap between formulations was quantified from the INCI lists using the Jaccard index, 100 × |A ∩ B|/|A ∪ B|, after harmonizing Aqua/Water and Aroma/Flavor naming. Pairwise overlap was 23.5% (Elmex–Eludril), 10.5% (Elmex–Listerine), 17.4% (Elmex–GUM), 8.3% (Eludril–Listerine), 18.5% (Eludril–GUM), and 18.5% (Listerine–GUM). This presence/absence metric includes water and excipients and ignores concentration. Because fluoride species, antiseptics, essential oils, solvents, and surfactants co-vary between products, the four mouthrinses represent complete-formulation factor levels rather than controls for individual active ingredients, whose effects cannot be isolated in this design.

Ingredients were classified qualitatively by function; undisclosed concentrations were not inferred from the INCI order. “Alcohol-free” denotes the absence of ethanol, not of polyols, and fluoride values refer to total fluoride. Elmex combines two fluoride sources with a nonionic solubilizer (PEG-40 hydrogenated castor oil), a biguanide antimicrobial, and a pH adjuster. Eludril combines two cationic antiseptics with humectants and a nonionic solubilizer and therefore represents a complete antiseptic formulation rather than an isolated chlorhexidine exposure. Listerine combines eucalyptol, methyl salicylate, thymol, and menthol with ethanol and poloxamer 407 as a solvent/solubilizer system, sorbitol as a humectant and sweetener, sodium saccharin as a sweetener, benzoic acid/sodium benzoate as a preservative system, and CI 42053 (Fast Green FCF) as a colorant. GUM combines fluoride and the cationic amphiphile LAE with nonionic solubilizers, humectants, and a citric acid/salt system. Buffer capacity and the concentrations of the essential oils, LAE, and other undisclosed ingredients were not measured.

Fusayama-type artificial saliva was prepared per liter of deionized water: NaCl, 0.40 g (6.84 mM); KCl, 0.40 g (5.37 mM); CaCl_2_·2H_2_O, 0.795 g (≈5.4 mM); NaH_2_PO_4_·2H_2_O, 0.78 g (≈5.0 mM); Na_2_S·9H_2_O, 0.005 g (≈0.02 mM); and urea, 1.00 g (16.65 mM). It was stored at 5 °C before use; the blank pH was 6.79–6.80. This electrolyte/urea medium contained no mucin, salivary proteins, enzymes, or microorganisms and does not reproduce whole human saliva.

### 2.3. Controlled Immersion Protocol

Each specimen was fully immersed in 15 mL of the assigned medium in an individual sterile, tightly sealed container, handled aseptically, and stored at 37 ± 1 °C in a forced-air laboratory incubator (SBS-LI-65, Steinberg Systems, Berlin, Germany) for 3, 7, 10, or 14 days. Sutures were exposed directly to the mouthrinse without prior conditioning in saliva. The medium was not renewed, so that renewal frequency was not introduced as an additional variable, and the net response of a closed material–medium system could be compared. This cumulative static model was designed for comparative material characterization, not to reproduce intermittent clinical rinsing. Without renewal, dissolved products may accumulate while ingredients may be depleted, adsorbed, or altered; particular effects may therefore be either amplified or attenuated relative to a constant chemical challenge. No intermittent-exposure or medium-renewal group was included.

### 2.4. Wet-State and Re-Dried Mass Measurements

Initial specimen mass (m_0_) was measured as supplied, without drying or conditioning, on a semi-micro analytical balance (XS205 DualRange, Mettler Toledo, Greifensee, Switzerland; readability 0.01 mg in the fine range); it is therefore an operational pre-exposure mass rather than a dry mass. After retrieval, absorbent paper was applied to the suture and replaced until a fresh piece remained dry, and the blotted specimen was weighed to obtain the wet-state mass (m_t). The same blotting procedure was applied to the re-drying cohort at Day 14. Apparent wet-state mass change and the wet-mass values paired with Fmax in the correlation analysis were obtained from the three tensile-tested specimens per condition.

Relative apparent mass change was calculated as Δm_app (%) = [(m_t − m_0_)/m_0_] × 100. Because a wet-state mass is compared with an as-supplied mass, Δm_app can combine liquid retention, swelling, adsorption, deposition, and material loss; it is therefore termed apparent wet-state mass change rather than polymer mass change or water uptake.

In the re-drying cohort (*n* = 5 per material–medium combination), specimens were rinsed with deionized water after Day 14 wet weighing and dried in a vacuum desiccator at 37 °C and below 10 mbar for 48–96 h until constant mass, defined as a difference of no more than 0.01 mg between consecutive weighings 24 h apart. Specimens were cooled to room temperature under dry conditions before weighing. The Day 14 net dry-mass change was calculated as Δm_dry (%) = 100 × (m_dry,14 − m_0_)/m_0_ and compares the dried post-exposure state with the unconditioned initial state. The wet-to-rinsed/dried mass difference (m_wet,14 − m_dry,14) was calculated separately; because rinsing preceded drying, it includes both drying-removable constituents and rinse-removable solutes or deposits. Neither quantity can separate polymer loss from residual nonvolatile material or from differences in initial moisture.

### 2.5. pH Measurements

Solution pH was measured with a SevenCompact Duo benchtop meter and an InLab Expert Pro-ISM electrode (Mettler Toledo, Greifensee, Switzerland; resolution 0.001 pH) at 25 °C with automatic temperature compensation, after three-point calibration with certified technical buffers (pH 4.01, 7.00, and 9.21) before each measurement series. Three technical readings, not independent replicates, were recorded per container. Post-exposure pH was measured in the containers of the three tensile-tested specimens per condition.

The material-related pH deviation, ΔpH_blank = pH_suture − pH_blank, was used to separate changes associated with the suture from time-dependent changes in the medium itself. Blank controls consisted of two containers of each medium (15 mL) without a suture, stored in the incubator under the same conditions as the specimen containers.

### 2.6. Mechanical Testing

Uniaxial tensile testing was performed on a universal testing machine (Z010, Zwick/Roell, Ulm, Germany) with an Xforce P 1 kN load cell. Each specimen end was secured around a shackle-type fixture with a 2–1–1 surgical knot, giving bilateral knot fixation and a free gauge length of 15 mm between fixation points. Specimens were tested in the blotted wet state immediately after weighing and mounting, within 2 min of blotting. Rupture at a fixture edge was predefined as an exclusion criterion; it did not occur, so all specimens were analyzed (*n* = 3 per condition). Break location, knot slippage, and failure mode (knot rupture versus loosening/slippage) were recorded from observations and force–crosshead displacement curves but were not analyzed as separate endpoints.

The crosshead was first moved at 10 mm/min to a preload of 3 N to remove slack and standardize alignment; the force reading was then zeroed, and loading continued at 50 mm/min until failure, with force and crosshead displacement recorded continuously. Reported Fmax values therefore represent the force increase above the 3 N preload, and the total load at failure corresponds to approximately Fmax + 3 N. For the weakest specimens (Fmax of approximately 3 N, e.g., PP in Eludril at Day 3), the preload amounted to about half of the total load and may have affected knot seating or caused pre-damage; this influence could not be quantified without a separate preload-sensitivity comparison.

The primary endpoint was maximum breaking force (Fmax, N), defined as the highest force recorded before failure after zeroing at the preload and reported as mean ± standard deviation (*n* = 3 per condition). Because Fmax was not normalized to cross-sectional area, it is a system-level property of the suture–knot assembly rather than a tensile strength; changes in Fmax may reflect changes in the polymer, filament load sharing, friction, or knot behavior.

### 2.7. Statistical Analysis

Fmax was analyzed by three-factor fixed-effects analysis of variance (ANOVA) with material (seven levels), mouthrinse (four levels), and exposure time (four levels), including all two-way interactions and the three-way interaction; the design was balanced, with *n* = 3 in each of the 112 cells. The Shapiro–Wilk test yielded *p* ≤ 0.05 in 8 of the 112 subgroups. With *n* = 3 per subgroup, normality testing has limited resolution; the parametric ANOVA is therefore interpreted with caution. Significance was set at *p* < 0.05. Effect size was expressed as partial η^2^, a conditional measure that depends on the model and is not additive across terms. Sample size was determined by material availability rather than an a priori power calculation.

Apparent wet-state mass change and pH were not included in the factorial ANOVA and are summarized descriptively. Exploratory Pearson and Spearman correlations between condition-level mean Δm_app and Fmax were interpreted as descriptive associations; because condition means may be confounded by material, formulation, and time, and no out-of-sample validation was performed, they do not constitute predictive models. Day 14 dry-mass data are reported as mean ± sample SD across five specimens, with percentage changes calculated per specimen before aggregation. Analyses were performed in Python 3.12.14 with NumPy 2.3.5 and SciPy 1.17.0.

For Day 14 re-drying endpoints, each mouthrinse was compared with artificial saliva for the same material using two-sided Welch *t*-tests (*n* = 5 per group), with percentage changes calculated per specimen before aggregation. Holm correction was applied separately to net dry-mass change and wet-to-rinsed/dried mass difference (28 contrasts each); p_H denotes adjusted *p*-values (α = 0.05). Fmax, wet-state mass, and pH comparisons with saliva remain descriptive. Technical pH readings were not treated as independent replicates (Supplementary Materials).

## 3. Results

### 3.1. pH of the Exposure Media

Initial pH differed between mouthrinses, from 4.11 for Listerine to 5.53 for GUM. Across all materials and time points, mean pH was 4.517 for Elmex, 5.398 for Eludril, 4.148 for Listerine, and 5.485 for GUM.

**Figure 3:**
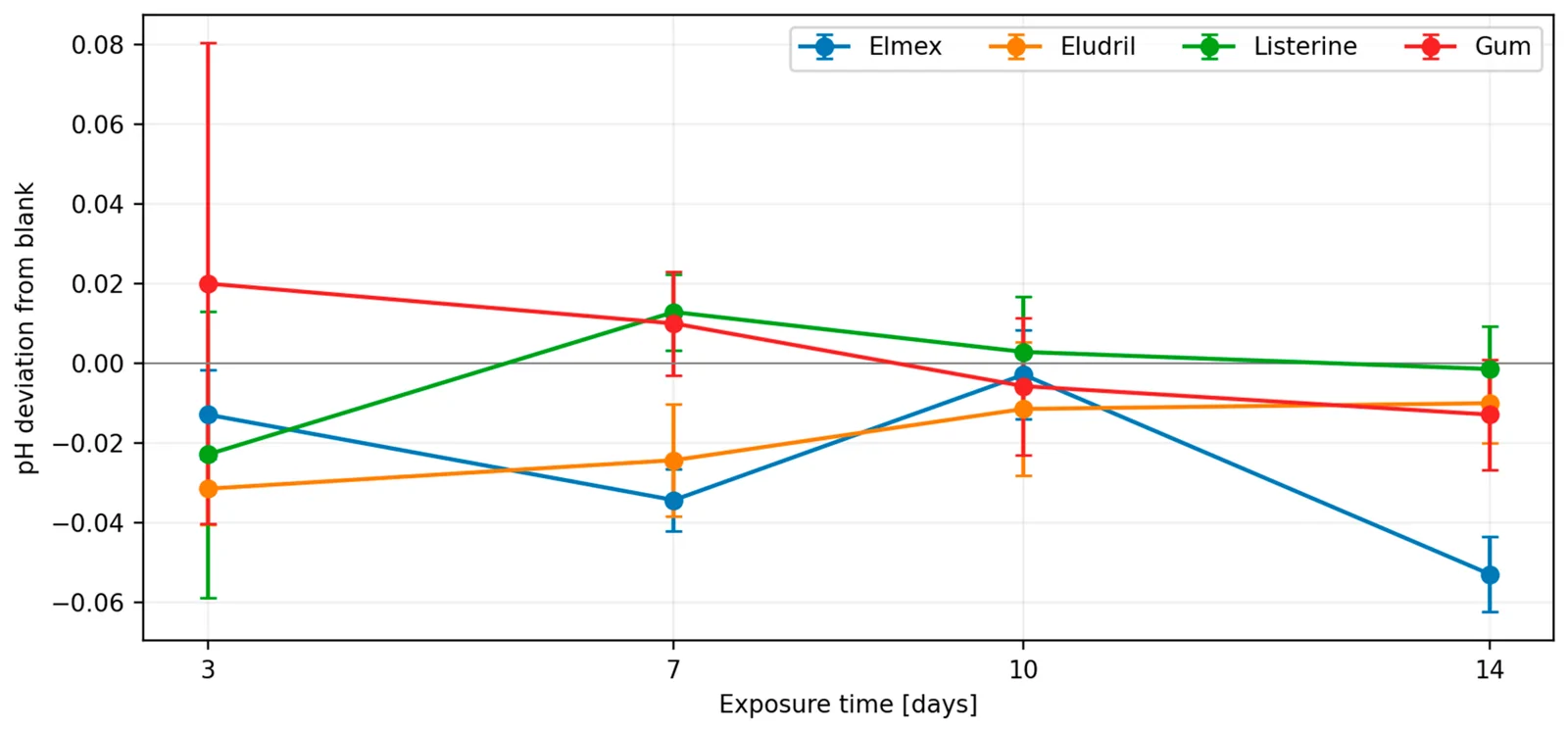
Difference between the pH of suture-containing solutions and the corresponding blank mouthrinses (ΔpH_blank), shown as mean ± SD across the seven suture materials at each time point.

The mean pH deviation from the corresponding blanks was −0.026 for Elmex, −0.019 for Eludril, −0.002 for Listerine, and +0.003 for GUM. The maximum absolute deviation was 0.10 pH units (Figure 3), indicating limited modification of the bulk acid–base environment in this model.

### 3.2. Maximum Breaking Force

Fmax differed markedly among materials. Three-factor ANOVA showed significant main effects of material, mouthrinse, and exposure time and significant two- and three-way interactions (all *p* < 0.001; Table 3). Material had the largest main effect (partial η^2^ = 0.957), followed by exposure time (partial η^2^ = 0.737). The interaction terms indicate that the effect of a mouthrinse or exposure duration depends on the suture material. These parametric results should be interpreted in light of the small subgroup sample size and normality-test findings.

Averaged across mouthrinses and exposure times, the mean Fmax was 11.84 N for PGLA, 9.12 N for NL, 7.66 N for PP, 7.36 N for SB, 6.61 N for PGA, 6.55 N for PB, and 6.14 N for SI. Averaged across materials and times, the mean Fmax was 8.10 N in Elmex, 8.13 N in Eludril, 7.08 N in Listerine, and 8.27 N in GUM; the material × mouthrinse interaction prevents interpreting these pooled means as a universal ranking.

### 3.3. Time-Dependent Mechanical Response

**Figure 4:**
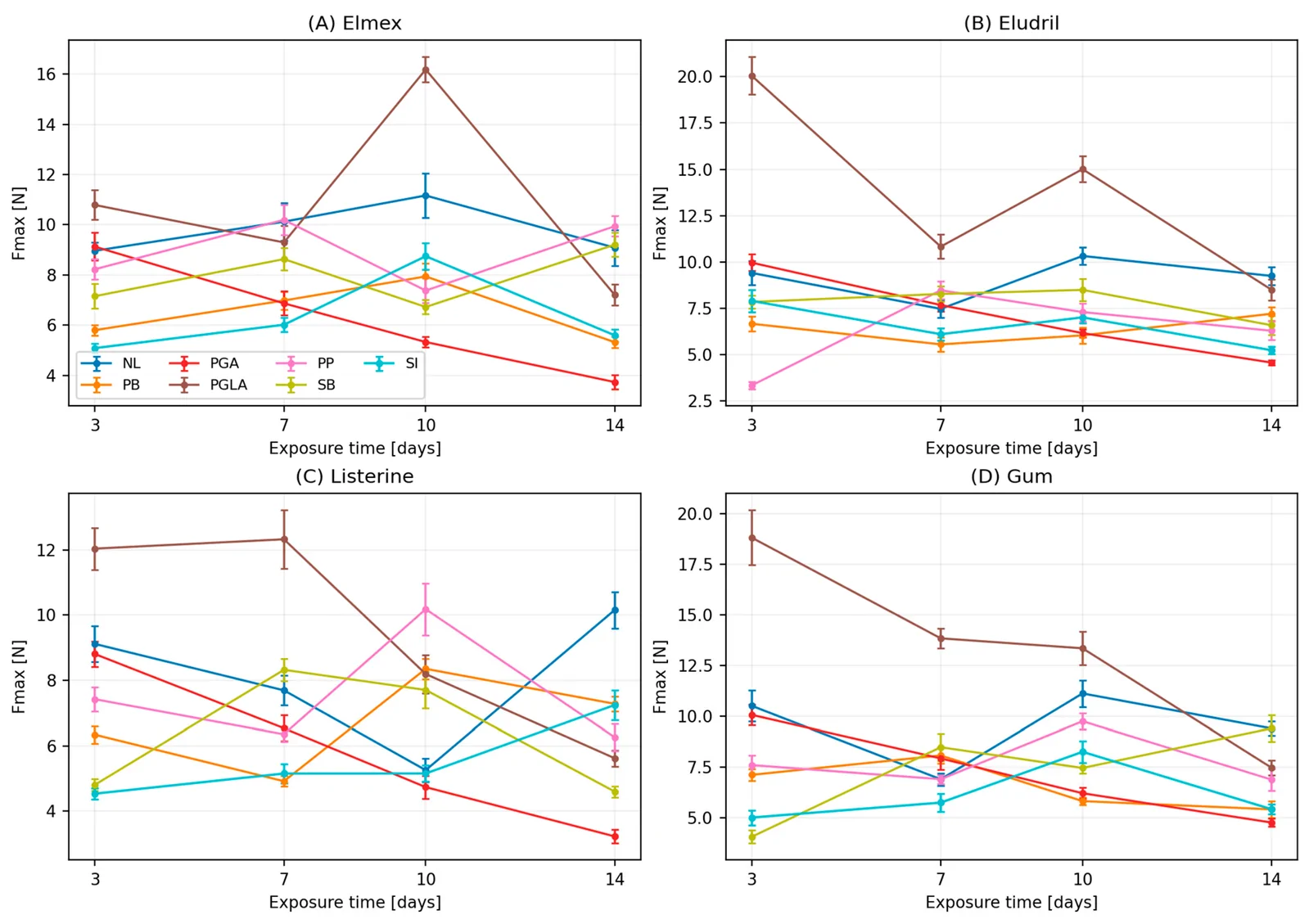
Time-dependent maximum breaking force (Fmax) of the seven suture materials in (**A**) Elmex, (**B**) Eludril, (**C**) Listerine, and (**D**) GUM. Data are mean ± SD (*n* = 3).

Pooled over materials and mouthrinses, the mean Fmax was 8.45 N on Day 3, 7.92 N on Day 7, 8.40 N on Day 10, and 6.82 N on Day 14. Averaged across mouthrinses, PGA and PGLA showed declines of 57.1% and 53.3% between Days 3 and 14. PB and NL changed by −2.7% and −0.3%, whereas PP, SB, and SI increased by 10.5%, 24.8%, and 4.3%, respectively (Table 4; Figure 4A–D).

PGLA in GUM changed from 18.80 ± 1.36 N to 7.46 ± 0.37 N (−60.3%); PGLA in Listerine changed from 12.04 ± 0.65 N to 5.62 ± 0.25 N (−53.3%). PGA in Elmex changed from 9.13 ± 0.55 N to 3.73 ± 0.29 N (−59.1%).

SI in Listerine changed from 4.53 ± 0.17 N to 7.24 ± 0.45 N (+59.8%); SB in GUM changed from 4.06 ± 0.32 N to 9.40 ± 0.66 N (+131.6%). For SB in Listerine at Day 14, the three recorded values were 4.424, 4.767, and 4.5943 N, giving Fmax = 4.5951 ± 0.1715 N. Opposing material–formulation trajectories underlie the interaction effects.

### 3.4. Apparent Wet-State Mass Changes

**Figure 5:**
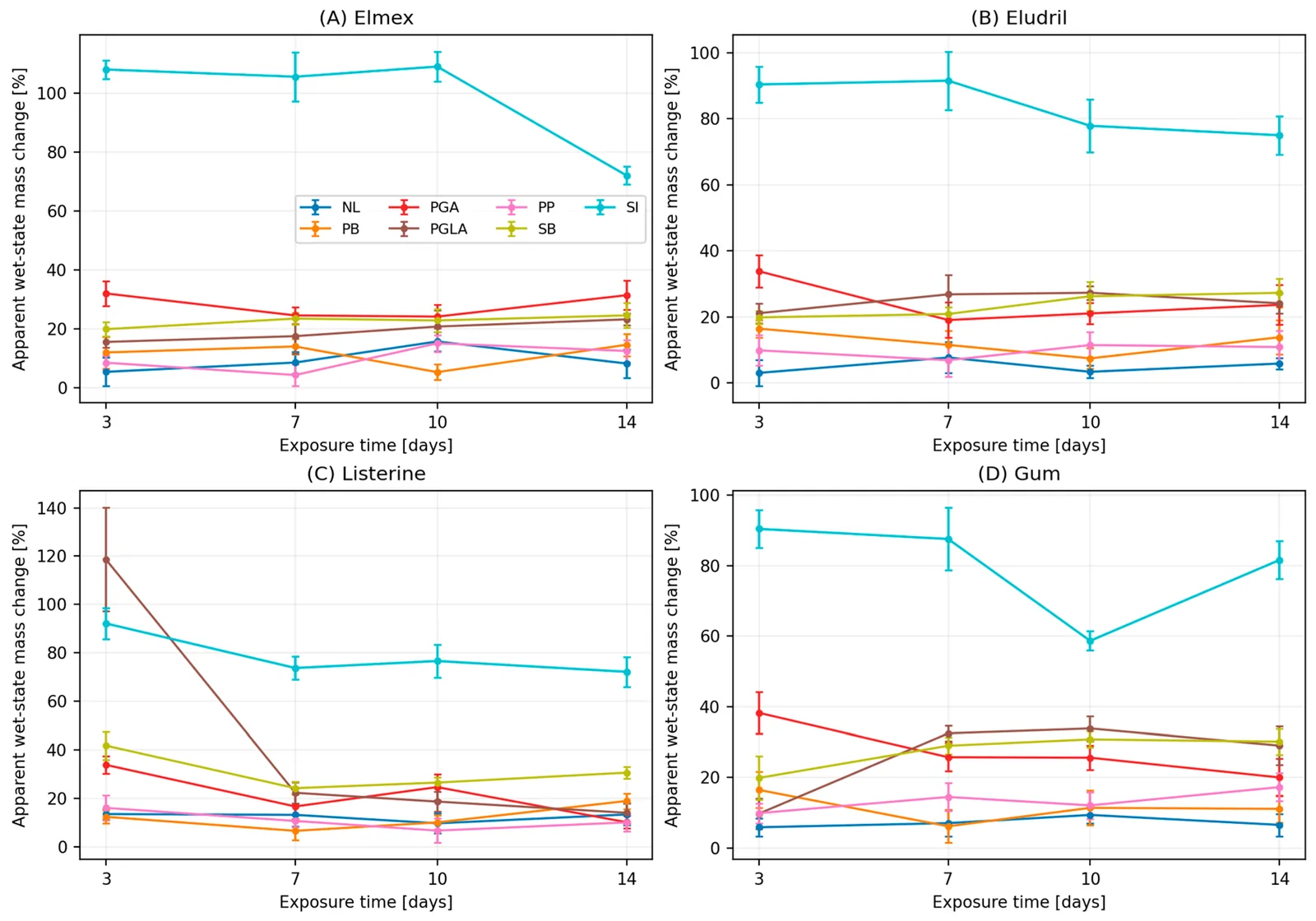
Apparent wet-state mass change of the seven suture materials in (**A**) Elmex, (**B**) Eludril, (**C**) Listerine, and (**D**) GUM, relative to the initial as-supplied mass. Values are mean ± SD (*n* = 3).

All materials showed positive apparent wet-state mass changes under at least some conditions, but the magnitude varied considerably (Table 5; Figure 5A–D). Averaged across mouthrinses and time points, Δm_app was largest for SI (85.1%), followed by PGLA (28.4%), SB (26.1%), PGA (25.3%), PB (11.8%), PP (11.0%), and NL (8.5%).

At Day 14, SI retained the largest response (72.0% in Elmex, 75.0% in Eludril, 72.2% in Listerine, and 81.6% in GUM), whereas NL changed by only approximately 5.9–13.3%, depending on the mouthrinse.

PGLA in Listerine showed a transient response: Δm_app was approximately 118.6% on Day 3 but 22.3%, 18.7%, and 14.0% on Days 7, 10, and 14. This pattern is inconsistent with simple progressive water uptake and may reflect early liquid retention or swelling followed by redistribution, desorption, material loss, or structural change.

Pooled across materials and times, Δm_app was similar between mouthrinses (Listerine 29.9%, Elmex 28.5%, GUM 27.5%, and Eludril 26.2%); between-material differences were therefore considerably larger than between-mouthrinse differences.

After rinsing and drying to constant mass at Day 14, net dry-mass changes were small compared with the wet-state response (Table 6). Negative values indicate a net loss and positive values a net gain of nonvolatile specimen-associated mass; neither identifies the species involved nor excludes simultaneous loss and deposition.

### 3.5. Relationship Between Apparent Mass Response and Mechanical Behavior

**Figure 6:**
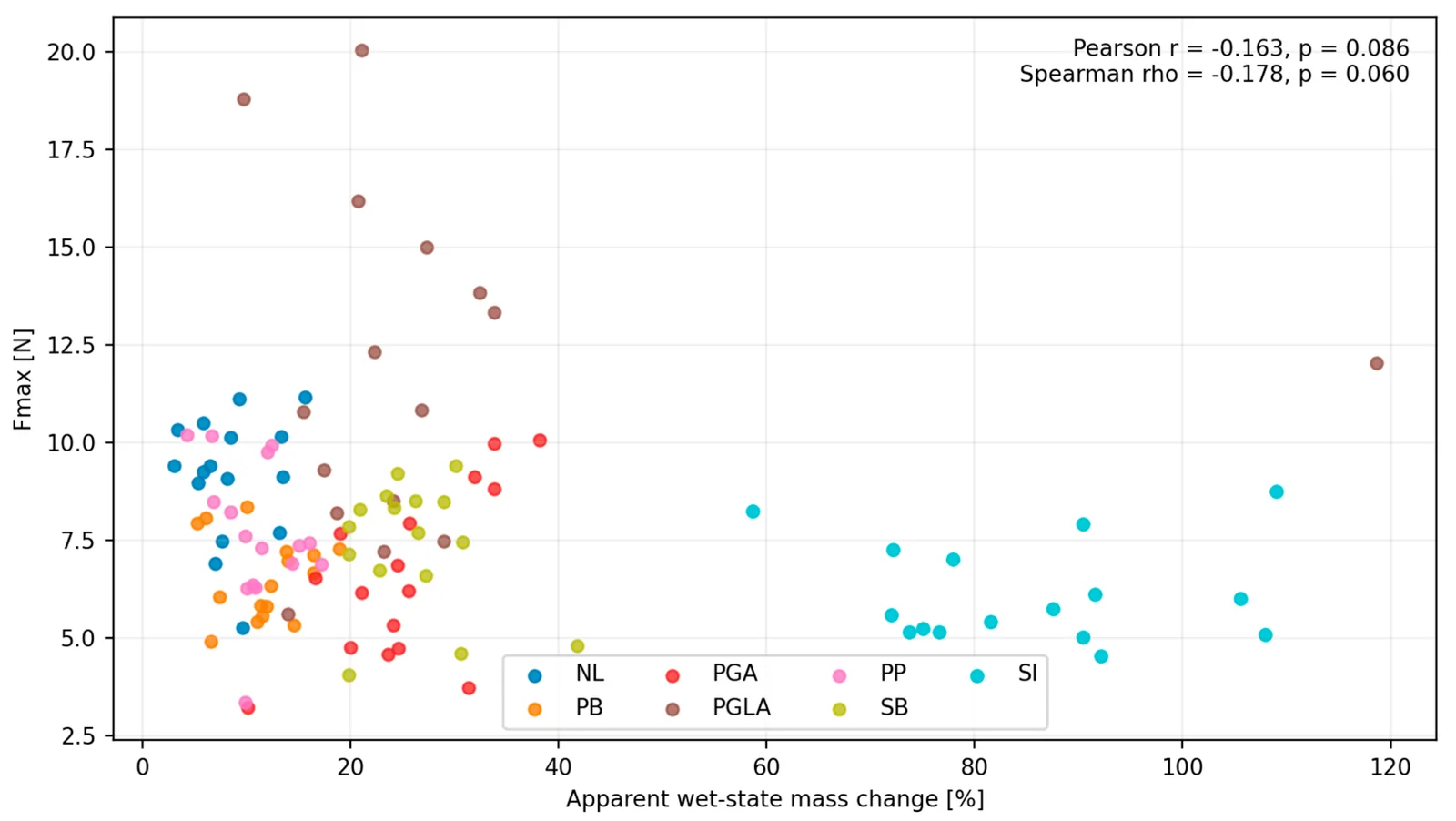
Relationship between apparent wet-state mass change and maximum breaking force (Fmax) across the 112 condition means.

Across the 112 material–mouthrinse–time condition means, the association between Δm_app and Fmax was weak (Figure 6). Pearson r = −0.163 (*p* = 0.086; R^2^ = 0.026) and Spearman ρ = −0.178 (*p* = 0.060). Neither pooled correlation reached *p* < 0.05. These findings do not establish the absence of an association within particular materials or formulations.

The contrast between SI and PGLA shows that wet-state mass and mechanical response need not follow parallel trajectories. These pooled correlations do not exclude associations within individual material–formulation combinations and do not validate prediction of Fmax from wet-state mass.

### 3.6. Artificial-Saliva Reference

In artificial saliva, Fmax trajectories were also material-dependent (Table 7). PGLA decreased from 18.03 N on Day 3 to 9.17 N on Day 14 (−49.1%), whereas SB increased from 6.19 to 13.11 N. Mechanical change during mouthrinse immersion therefore cannot automatically be attributed to the formulation.

Wet-state mass gains in artificial saliva persisted at all four time points, and Day 14 re-drying again yielded small net dry-mass changes (Table 8). Specimen-containing saliva pH ranged from 6.75 to 6.82, with a maximum absolute deviation of 0.04 pH units from the time-matched blank.

### 3.7. Comparisons with Artificial Saliva

At Day 3, the mean Fmax averaged across mouthrinses was lower than the saliva reference for PGA (9.49 versus 11.18 N) and PGLA (15.42 versus 18.03 N). At Day 14, the mouthrinse means for PGA (4.07 N), PGLA (7.20 N), PB (6.31 N), and SB (7.45 N) were below the corresponding saliva means (10.91, 9.17, 6.49, and 13.11 N), whereas NL, PP, and SI were above their respective references (Table 4 and Table 7). These are descriptive complete-medium comparisons that average over different formulation-specific effects.

In the re-drying cohort (*n* = 5 per group), 1 of 28 Day 14 mouthrinse–saliva contrasts had p_H < 0.05 for net dry-mass change (Table 9) and 4 of 28 for the wet-to-rinsed/dried mass difference. These complete-medium contrasts do not identify chemical residues or polymer loss; the wet-to-rinsed/dried difference includes material removed during rinsing as well as drying.

## 4. Discussion

The results show that suture performance under mouthrinse exposure is governed by material–environment coupling rather than by a uniform “degradation” response. Material type had the largest main effect, and the large material × mouthrinse, material × time, and three-way interactions show that the influence of solution chemistry and exposure duration depends strongly on polymer and filament architecture.

This agrees with studies of aqueous aging that report substantial differences in maximum load, deformation, stiffness, and failure behavior among commercial sutures under standardized conditions [5,6,7,8,9,10], and distinct time-dependent trajectories for PGA, polyglactin, polydioxanone, polypropylene, nylon, silk, and polyester in artificial saliva and other physiological media [8,11,12,14,15,16,29,30]. The present study extends this work to commercial oral-care formulations by combining a balanced factorial design with mechanical, gravimetric, and pH endpoints and a time-matched saliva reference.

### 4.1. Material-Specific Mechanical Response

PGLA had the highest overall mean Fmax but declined by 53.3% between Days 3 and 14 when averaged across mouthrinses; PGA declined by 57.1%. High early breaking force therefore does not guarantee sustained mechanical support. PGLA also declined in artificial saliva (−49.1%), showing that its temporal loss was not exclusive to mouthrinse exposure. At Day 14, the mean Fmax for PGLA was 7.20 N across mouthrinses and 9.17 N in saliva; PGA was 4.07 and 10.91 N, respectively. These descriptive differences do not isolate the responsible formulation components.

This response is compatible with the hydrolytic susceptibility of synthetic absorbable polymers such as polyglactin, polyglycolic acid, and polydioxanone, in which aqueous exposure promotes water penetration, chain scission, molecular-weight reduction, and progressive loss of mechanical integrity at a rate that depends on polymer structure, crystallinity, filament construction, and the chemical environment [11,12,13,14,15,16,31,32]. Pronounced time-dependent losses of polyglactin- and PGA-based sutures have also been reported in artificial saliva, buffered fluids, and oral-care solutions [8,12,17,19].

SB, PP, and SI showed net increases in mean Fmax of 24.8%, 10.5%, and 4.3%, respectively, whereas PB and NL changed little when averaged across mouthrinses (Table 4). Increases in the force of a suture–knot assembly should not be interpreted as polymer strengthening. Wet conditioning can modify filament packing, coating behavior, friction, and knot seating, redistributing stress within the assembly. The present endpoints cannot separate these processes.

### 4.2. Mouthrinse Effects Depend on the Suture Material

Pooled across materials and times, the mean Fmax was lowest in Listerine and highest in GUM, but the material × mouthrinse interaction makes an overall ranking inappropriate: individual material–formulation pairs followed different trajectories.

This agrees with earlier mouthrinse studies. Alsarhan et al. reported material- and gauge-dependent responses of absorbable sutures to chlorhexidine and Listerine [17]; Abullais et al. found that four mouthwashes affected Vicryl, PTFE, polypropylene, and silk differently [18]; and work combining chlorhexidine exposure with thermal cycling showed that the response of multifilament sutures cannot be generalized across materials [19].

Clinically, a mouthrinse should therefore not be regarded as universally compatible or incompatible with sutures; the relevant unit of comparison is the material–solution combination over the period in which wound support is required.

### 4.3. Apparent Wet-State Mass Response

Parallel assessment of mechanical and gravimetric response was a key feature of the design. Δm_app ranged from approximately 85% for SI to 8.5% for NL, consistent with differences in filament architecture, porosity, capillarity, surface chemistry, and polymer affinity for aqueous media [1,2,10].

The high response of silk is plausible for a natural multifilament with a large surface area and capillary spaces between filaments, whereas monofilaments such as nylon and polypropylene offer few interfilament spaces for retained liquid. However, the gravimetric endpoint cannot distinguish absorption into the polymer matrix from liquid retained between filaments or on the surface.

The non-monotonic response of PGLA in Listerine, with a large early gain followed by a substantial decrease, illustrates this limitation. It may reflect an early hydration-dominated phase followed by redistribution of retained solution, desorption, dissolution of surface components, or degradation-related material loss; direct chemical or microscopic methods are needed to distinguish these mechanisms. Day 14 re-drying showed that most of the wet-state gain was removed by rinsing and drying, leaving net dry-mass changes of only a few percent.

### 4.4. Association Between Wet-State Mass and Mechanical Response

The weak pooled correlation between Δm_app and Fmax describes the present dataset but is not a mechanistic test. Differences in polymer identity, construction, knot behavior, and exposure time may obscure within-material associations, so the result does not show that hydration is mechanically irrelevant or that mass could not contribute to a suitably specified and validated predictive model.

SI showed the largest mass response without a progressive 14-day decrease in Fmax, whereas PGLA lost Fmax despite a lower mean mass response. Consistent with the literature, a polymer may retain a large amount of liquid without major chain scission, whereas a hydrolytically susceptible polyester may lose molecular weight and mechanical function with only a modest net wet-state mass change. Because mechanical behavior also depends on filament architecture, knot mechanics, local stress concentrations, polymer orientation, and coating changes, Δm_app should be treated as a complementary gravimetric indicator rather than a surrogate for retained mechanical function.

### 4.5. Bulk pH and Formulation Chemistry

Although the initial pH of the mouthrinses differed by approximately 1.4 units, the sutures produced only small shifts relative to the blanks, indicating that they did not substantially modify the bulk acid–base environment within 14 days. The acidic initial range (pH 4.11–5.53) does not by itself indicate erosive risk: the often-cited value of approximately 5.5 is not a universal safety threshold, because mineral dissolution also depends on saturation, calcium/phosphate and fluoride availability, buffering, titratable acidity, contact time, and salivary clearance [33]. Titratable acidity and dental hard-tissue outcomes were not measured, so these data cannot support recommendations to avoid a particular rinse.

Small bulk pH changes do not exclude local interactions within hydrated polymer regions or at the knot interface, and several formulation-related mechanisms are plausible. Nonionic solubilizers and amphiphiles can modify wetting and access to spaces between braided filaments, while humectants and dissolved solutes can affect the amount and composition of retained liquid, so a wet-mass increase may contain both solvent and solute contributions; polyols, surfactants, salts, and other nonvolatile residues may contribute to the re-dried mass. Cationic ingredients (chlorhexidine, cetylpyridinium, biguanide species, and LAE) could adsorb on accessible charged or polar sites, and solvents could alter access to coatings or polymer regions. Hydration of polyamides may modify deformation and knot seating, and altered wetting or interfilament friction could change load sharing and Fmax without changing intrinsic polymer strength, whereas hydrolysis remains a plausible concurrent process in absorbable polyester-based sutures [11,12,13,14,15,16].

None of these processes were demonstrated. Surface composition, porosity, roughness, zeta potential, adsorption, residues, and molecular weight were not measured, and the presence of citrate, lactate, benzoate, or fluoride does not by itself indicate catalytic degradation. Because the design compares complete commercial formulations with shared excipients and undisclosed concentrations, no observed change can be assigned to pH, ethanol, an antiseptic, or a surfactant individually; ingredient-defined control solutions and residue characterization are needed to test these explanations.

### 4.6. Bioengineering and Translational Relevance

A surgical suture must provide time-matched mechanical function: load-bearing capacity should persist while tissue strength is insufficient and may decline once wound stability no longer depends on the device. Because retention trajectories differed substantially between polymers, ranking materials by a single early Fmax value can be misleading. Material selection for oral surgery should integrate polymer chemistry, filament architecture, absorbability, knot behavior, and the expected chemical environment rather than rely on nominal material class.

The data also do not support a universal ranking of mouthrinses: compatibility is specific to the material–solution pair and evolves with exposure time. Designing sutures for the oral cavity therefore requires attention not only to dry-state mechanical properties but also to the stability of the material–environment interface under oral-care conditions.

### 4.7. Limitations and Future Engineering Work

The static, unreplenished exposure model does not reproduce intermittent clinical rinsing, and the lack of renewal can alter ingredient availability and the accumulation of released products in either direction. Sutures were not preconditioned in saliva, so pellicle formation and its effects on wetting, adsorption, diffusion, and friction were not represented; the separate saliva group provides a reference for aqueous/electrolyte exposure but not sequential salivary conditioning followed by mouthrinse exposure. Salivary proteins, enzymes, microorganisms and biofilms, temperature fluctuations, tissue movement, and masticatory loading were absent, and their net effects cannot be inferred from this experiment.

Each mechanical condition included three specimens from commercially packaged sutures of the same manufacturing batch per suture type. Multiple boxes were used, but package identity was not modeled, and between-batch reproducibility was not assessed. Although within-condition dispersion was relatively small, the modest sample size limits the precision of individual pairwise differences and higher-order interactions. Mechanical comparisons with artificial saliva were descriptive, and the re-dried mass data derive from a separate *n* = 5 cohort that was not tensile-tested and are available for Day 14 only.

Fmax describes the complete suture–knot system rather than an intrinsic tensile property: cross-sectional area was not used to calculate stress, and failure location and knot security were not analyzed as separate endpoints. Because the force signal was zeroed at the 3 N preload, relative changes in Fmax are larger than the corresponding changes in total load at failure would be, and the preload may have influenced knot seating in the weakest specimens. In addition, percentage changes are relative to Day 3 rather than to the unexposed state.

Δm_app integrates liquid retention, hydration, swelling, adsorption, deposition, and material loss. Day 14 re-drying separates the wet-to-rinsed/dried difference from net dry-mass change but does not resolve these processes chemically: nonvolatile residues can mask material loss, a dry-mass decrease does not by itself prove backbone degradation, and the wet-to-rinsed/dried difference is not pure water uptake. Comparisons with saliva represent complete-medium differences, because the near-neutral reference differs from the acidic rinses in both pH and composition.

Chemical and morphological changes were not characterized. Scanning electron microscopy would establish whether morphological changes accompany the measured responses, and FTIR or Raman spectroscopy, thermal analysis, and molecular-weight determination would be required to identify chemical transformations. Future work should also derive displacement at Fmax, displacement at failure, apparent stiffness, and work to failure from the recorded force–crosshead displacement traces and combine them with surface characterization to build a more mechanistic material–environment response map.

## 5. Conclusions

Continuous static immersion in commercial mouthrinses produced material-, formulation-, and time-dependent responses in the maximum breaking force of the suture–knot system. All main effects and interactions were significant in the factorial ANOVA, interpreted with caution because of *n* = 3 per condition and the normality-test findings. Mean Fmax declined by 57.1% for PGA and 53.3% for PGLA between Days 3 and 14 when averaged across mouthrinses, whereas other materials showed increases or relatively small net changes. The decline in PGLA in artificial saliva showed that temporal loss was not exclusive to mouthrinse exposure. Apparent wet-state mass varied widely and showed a weak, statistically nonsignificant pooled association with Fmax; rinsing and drying removed much of the wet-state gain, and changes in bulk pH were limited. These findings apply to unreplenished immersion at 37 °C without salivary preconditioning and cannot be directly extrapolated to intermittent clinical use. Direct chemical and surface characterization and more representative exposure models are needed to establish mechanisms and clinical relevance.

## Figures and Tables

**Table 1:** Characteristics of the investigated surgical suture materials (nominal length 75 cm; USP size 4-0).

Code	Product Code	Material	Origin	Absorbability	Structure
NL	NL40F0619	Nylon 66	Synthetic	Non-absorbable	Monofilament
PP	PP40F0619	Polypropylene	Synthetic	Non-absorbable	Monofilament
PB	PB40F0619	Polyester	Synthetic	Non-absorbable	Multifilament
PGLA	PGLA40F0619	Polyglactin	Synthetic	Absorbable	Multifilament
PGA	PGA40F0619	Polyglycolic acid	Synthetic	Absorbable	Multifilament
SI	SI40HR20	Silk	Natural	Non-absorbable	Multifilament
SB	SB40HS20	Polyamide	Synthetic	Non-absorbable	Core–sheath/multifilament

**Table 2:** Commercial formulations used as exposure media: recorded INCI lists, declared active concentrations, measured initial pH, and batch information. Undisclosed concentrations were not inferred from the ingredient order.

Commercial Product/Identification	INCI Composition/Formulation Characteristics	Initial pH	Batch/Expiry
Elmex Sensitive, 400 mL Colgate-Palmolive Manufacturing (Poland), Świdnica, Poland	Alcohol- and colorant-free fluoride mouthrinse. INCI: aqua, PEG-40 hydrogenated castor oil, olaflur, aroma, potassium acesulfame, sodium fluoride, polyaminopropyl biguanide, hydrochloric acid. Contains olaflur (amine fluoride) and sodium fluoride; total fluoride content: 250 ppm F. Individual fluoride-source and excipient concentrations were not disclosed.	4.52	LOT 6019P1111; EXP 06/2028
Eludril Care, 500 mL Pierre Fabre Oral Care/Pierre Fabre Médicament, Lavaur, France	Alcohol-free antiplaque mouthrinse containing 0.05% chlorhexidine digluconate and 0.05% cetylpyridinium chloride. INCI: water (aqua), propylene glycol, xylitol, glycerin, cetylpyridinium chloride, chlorhexidine digluconate, flavor (aroma), lactic acid, limonene, PEG-40 hydrogenated castor oil, potassium acesulfame, Red 40 (CI 16035), sodium propionate. No fluoride ingredient is listed. Excipients include humectants, a solubilizer, flavoring, sweetener, acidifier, preservative, and colorant; their concentrations were not disclosed.	5.43	LOT 5G3GD; MFG 12/2025; EXP 12/2028
Listerine Cool Mint, 500 mL Kenvue/JNTL Consumer Health, EU formulation, Warsaw, Poland	Alcohol-containing essential-oil mouthrinse. INCI: aqua, alcohol, sorbitol, poloxamer 407, benzoic acid, sodium saccharin, eucalyptol, methyl salicylate, thymol, sodium benzoate, menthol, aroma, CI 42053. Ethanol content: 21.6%. Individual essential-oil concentrations were not recorded. No fluoride ingredient is listed.	4.11	Batch/production code: 100830 01:39 11; PAO: 12 months
GUM ActiVital Fresh Mint, 500 mL Sunstar Europe S.A./Sunstar Etoy, Switzerland	Alcohol-free fluoride mouthrinse (248 ppm F^−^) with ethyl lauroyl arginate (LAE). INCI: aqua, glycerin, propylene glycol, PEG-40 hydrogenated castor oil, poloxamer 407, isomalt, sodium benzoate, ethyl lauroyl arginate HCl, aroma, sucralose, sodium fluoride, bisabolol, punica granatum fruit extract, ubiquinone, zingiber officinale root extract, potassium sorbate, sodium citrate, CI 42053, citric acid. LAE and other non-fluoride ingredient concentrations were not disclosed.	5.53	LOT X01; EXP 02/2027

**Table 3:** Three-factor analysis of variance for maximum breaking force (Fmax).

Effect	df	F	*p*-Value	Partial η^2^
Material	6, 224	828.58	<0.001	0.957
Mouthrinse	3, 224	108.99	<0.001	0.593
Time	3, 224	209.24	<0.001	0.737
Material × Mouthrinse	18, 224	40.34	<0.001	0.764
Material × Time	18, 224	142.02	<0.001	0.919
Mouthrinse × Time	9, 224	19.98	<0.001	0.445
Material × Mouthrinse × Time	54, 224	38.93	<0.001	0.904

**Table 4:** Mean Fmax at Days 3 and 14, averaged across the four mouthrinses.

Material	Day 3 Fmax [N]	Day 14 Fmax [N]	Change
SI	5.63	5.88	+4.3%
SB	5.97	7.45	+24.8%
PGA	9.49	4.07	−57.1%
PB	6.48	6.31	−2.7%
PP	6.65	7.34	+10.5%
PGLA	15.42	7.20	−53.3%
NL	9.50	9.47	−0.3%

**Table 5:** Mean apparent wet-state mass change at Days 3 and 14, averaged across the four mouthrinses.

Material	Day 3 Δm_app [%]	Day 14 Δm_app [%]
SI	95.2	75.2
SB	25.3	28.1
PGA	34.5	21.3
PB	14.3	14.6
PP	11.1	12.7
PGLA	41.2	22.6
NL	6.9	8.5

**Table 6:** Day 14 net dry-mass change relative to the initial as-supplied mass (Δm_dry) and wet-to-rinsed/dried mass difference (wet mass minus re-dried mass; not chemically specific). Values are mean ± sample SD of specimen-level values (*n* = 5 per condition).

Mouthrinse	Suture	Δm_dry [%]	Wet-to-Rinsed/Dried Difference [mg]
ELMEX	PGA	−4.00 ± 1.37	2.655 ± 0.148
	PGLA	−3.99 ± 1.38	2.760 ± 0.132
	NL	−0.78 ± 0.32	0.540 ± 0.215
	PB	0.77 ± 0.37	0.950 ± 0.115
	PP	0.30 ± 0.31	0.610 ± 0.128
	SB	−0.27 ± 0.38	1.935 ± 0.152
	SI	−6.23 ± 0.34	5.440 ± 0.099
ELUDRIL	PGA	−2.80 ± 1.40	1.985 ± 0.204
	PGLA	−3.99 ± 1.39	2.855 ± 0.072
	NL	−0.28 ± 0.33	0.370 ± 0.025
	PB	−0.40 ± 0.35	0.980 ± 0.211
	PP	−0.70 ± 0.34	0.580 ± 0.186
	SB	0.24 ± 0.39	2.110 ± 0.165
	SI	−2.06 ± 0.34	5.360 ± 0.281
GUM	PGA	−3.60 ± 1.39	1.770 ± 0.162
	PGLA	−4.09 ± 1.38	3.360 ± 0.238
	NL	−0.28 ± 0.32	0.410 ± 0.113
	PB	−0.83 ± 0.35	0.820 ± 0.133
	PP	−0.30 ± 0.33	0.880 ± 0.135
	SB	−0.66 ± 0.38	2.400 ± 0.126
	SI	−2.20 ± 0.33	5.825 ± 0.249
LISTERINE	PGA	−3.74 ± 1.39	1.040 ± 0.074
	PGLA	−2.61 ± 1.40	1.690 ± 0.094
	NL	−0.45 ± 0.32	0.830 ± 0.196
	PB	1.20 ± 0.38	1.220 ± 0.073
	PP	−0.50 ± 0.32	0.530 ± 0.118
	SB	−1.30 ± 0.37	2.490 ± 0.016
	SI	−1.34 ± 0.34	5.110 ± 0.311

**Table 7:** Maximum breaking force in artificial saliva (mean ± sample SD, *n* = 3). Percentage change compares Day 14 with Day 3, not with the unexposed state.

Suture	Day 3 [N]	Day 7 [N]	Day 10 [N]	Day 14 [N]	Change [%]
PGA	11.18 ± 0.34	11.94 ± 0.36	11.06 ± 0.33	10.91 ± 0.33	−2.5
PGLA	18.03 ± 0.54	13.94 ± 0.42	14.21 ± 0.43	9.17 ± 0.28	−49.1
NL	9.73 ± 0.29	8.20 ± 0.25	8.72 ± 0.26	8.63 ± 0.26	−11.3
PB	6.60 ± 0.20	6.41 ± 0.19	7.78 ± 0.23	6.49 ± 0.19	−1.5
PP	6.49 ± 0.19	7.03 ± 0.21	8.44 ± 0.25	7.28 ± 0.22	+12.3
SB	6.19 ± 0.19	8.78 ± 0.26	6.69 ± 0.20	13.11 ± 0.39	+112.0
SI	6.55 ± 0.20	6.41 ± 0.19	7.84 ± 0.24	5.57 ± 0.17	−15.0

**Table 8:** Gravimetric response in artificial saliva. Wet-state changes (*n* = 3) were calculated from the recorded as-supplied and wet masses; Day 14 dry-mass change and wet-to-rinsed/dried mass difference are mean ± sample SD (*n* = 5, re-drying cohort).

Suture	Wet Δm D3 [%]	D7 [%]	D10 [%]	D14 [%]	Dry Δm D14 [%]	Wet–Dry Difference [mg]
PGA	25.4	18.1	22.4	20.6	−3.53 ± 1.66	1.862 ± 0.130
PGLA	38.1	20.5	22.6	20.6	−3.67 ± 1.42	2.666 ± 0.111
NL	6.9	7.7	9.0	7.7	−0.45 ± 0.56	0.537 ± 0.138
PB	12.1	9.3	8.3	12.1	0.19 ± 0.86	0.993 ± 0.162
PP	16.0	8.2	9.2	13.2	−0.30 ± 0.50	0.650 ± 0.147
SB	26.3	24.6	24.8	26.3	−0.50 ± 0.90	2.234 ± 0.171
SI	78.2	76.4	63.7	74.4	−2.96 ± 1.03	5.434 ± 0.246

**Table 9:** Day 14 contrasts in net dry-mass change versus artificial saliva: difference in percentage points (p_H; Holm correction across 28 contrasts). Initial mass was measured as supplied. Positive values indicate a greater net dry-mass change in mouthrinse.

Suture	Elmex	Eludril	Listerine	GUM
PGA	−0.47 (1.0000)	+0.73 (1.0000)	−0.20 (1.0000)	−0.07 (1.0000)
PGLA	−0.32 (1.0000)	−0.32 (1.0000)	+1.06 (1.0000)	−0.42 (1.0000)
NL	−0.33 (1.0000)	+0.17 (1.0000)	0.00 (1.0000)	+0.17 (1.0000)
PB	+0.58 (1.0000)	−0.58 (1.0000)	+1.01 (1.0000)	−1.02 (1.0000)
PP	+0.60 (1.0000)	−0.40 (1.0000)	−0.20 (1.0000)	0.00 (1.0000)
SB	+0.22 (1.0000)	+0.73 (1.0000)	−0.80 (1.0000)	−0.16 (1.0000)
SI	−3.28 (0.0348)	+0.90 (1.0000)	+1.61 (0.5940)	+0.75 (1.0000)

## Data Availability

The data supporting the reported results, including raw force–crosshead displacement records, are available from the corresponding author upon reasonable request.

## Supplementary Materials

The following supporting information can be downloaded at: https://www.mdpi.com/article/10.3390/bioengineering13101178/s1, Table S1: Fmax; Table S2: Wet mass; Table S3: Redrying; Table S4: pH and mass summaries; Table S5: Initial diameter.
